# Parkinsonism after ventriculoperitoneal shunt for hydrocephalus

**DOI:** 10.1186/s12883-023-03064-2

**Published:** 2023-01-24

**Authors:** Yue Zhang, Bi W. Chen, Wei Mao, Feng Y. Wu, Yan Zhang

**Affiliations:** 1 Department of Neurology, Xuanwu Hospital, Capital Medical University, Beijing, 100053 China; 2 Institute Of Sleep And Consciousness Disorders, Beijing Institute of Brain Disorders, Collaborative Innovation Center for Brain Disorders, Capital Medical University, Beijing, 100053 China

**Keywords:** Parkinsonism, Ventriculoperitoneal shunt, Hydrocephalus, Complication, Case report

## Abstract

**Background:**

Parkinsonism after ventriculoperitoneal shunt in patients with hydrocephalus is a rare and profound complication that is often misdiagnosed, causing treatment to be delayed. To date, the characteristics of this disease have not been well described and summarized. Here, we report a rare case of parkinsonism after ventriculoperitoneal shunt; symptoms were aggravated by antipsychotic drugs but showed a good response to Madopar. Such cases have rarely been reported previously.

**Case presentation:**

A 44-year-old man presented with parkinsonism, bilateral pyramidal tract signs, and oculomotor impairment four years after a successful ventriculoperitoneal shunt for idiopathic aqueduct stenosis resulting in obstructive hydrocephalus. Brain magnetic resonance imaging and computed tomography showed fluctuations in the lateral ventricle and the third ventricle without any intervention. The patient’s condition was aggravated by antipsychotic drugs but showed a good response to Madopar.

**Conclusion:**

This observation suggests that parkinsonism in this patient was caused by reversible dysfunction of the presynaptic nigrostriatal dopaminergic pathway due to fluctuations in the lateral ventricle, representing the first hit to the dopaminergic signalling pathway, and antipsychotic drugs had an antagonistic effect on dopamine D2 receptors, representing the second hit. In addition, we summarize the pathophysiological mechanisms, clinical manifestations, treatments, and prognoses of this complication in 38 patients who met the inclusion criteria in 24 previous studies to increase neurologists’ understanding of the disease.

## Background

Hydrocephalus is a common adult neurosurgical condition characterized by overaccumulation of cerebrospinal fluid (CSF) in the ventricles, which can affect cognitive function, vision, appetite, and cranial nerve function. The current treatment for hydrocephalus uses ventriculoperitoneal (VP) shunts with valves to redirect CSF from the ventricles into the peritoneum [1]. Unfortunately, several complications can occur due to shunt technology, including infection, catheter occlusion, and overdrainage or underdrainage of CSF due to valve malfunction [2]. However, parkinsonism is rare after single or multiple VP shunt in patients with hydrocephalus. The mechanisms that are widely accepted at present involve impairments in two pathways: the presynaptic nigrostriatal dopaminergic pathway and the cortico-basal ganglia loop. Different therapeutic strategies are used for the different pathways involved. The former requires high doses of dopaminergic drugs, but endoscopic third ventriculostomy (ETV) is more effective for the latter. Early diagnosis and pharmacological treatment are crucial for a patient’s recovery; therefore, it is essential that neurologists pay attention to this rare but devastating postoperative complication. This paper presents the case of a middle-aged man with obstructive hydrocephalus due to idiopathic midbrain aqueduct stenosis who developed parkinsonism and akinetic mutism 4 years after right-sided VP shunt. Along with this case, we summarize the literature to improve the understanding of this disease within the field and to clarify the most effective direction of treatment.

## Case presentation

In 2016, a 44-year-old male presented with sudden intermittent headache and dizziness and progressive loss of vision lasting for 3 months. He was diagnosed with obstructive hydrocephalus due to idiopathic aqueduct stenosis and underwent a right VP shunt procedure on September 10, 2016. His symptoms were completely relieved after the operation.

On February 15, 2021, he developed stiff facial expressions. After two weeks, he gradually developed bradykinesia and body stiffness. At that time, the patient’s family complained that he was depressed because of recent family conflict. A psychiatrist diagnosed him with catatonic syndrome. The patient subsequently started paroxetine and mirtazapine treatment. One month later, his symptoms began gradually intensifying, and he developed significant limitation of vertical ocular movements, parkinsonian symptoms of cogwheel rigidity and shaking, limb bradykinesia, and difficulty turning over in bed. In addition, his voice became slow and soft. Most of the time, he was silent and presented memory loss. Given the possibilities of parkinsonism, autoimmune encephalitis, and depressive stupor, the patient received duloxetine, clonazepam, donepezil, amantadine, ganciclovir, and methylprednisolone.

After two months of treatment, the patient became bedridden and was eventually admitted to our hospital. He gradually developed drowsiness, had to be fed through a nasogastric tube and needed tracheal intubation on April 19, 2021. Neurological examination showed high myodynamia, bilateral pyramidal signs, and hyperreactive deep-tendon reflexes. Madopar (125 milligrams (mg) ter in die) was administered to improve parkinsonism. Duloxetine (20 mg quaque die), clonazepam (2 mg bis in die) and olanzapine (5 mg quaque nocte) were given to treat depressive stupor, all without effect. Considering that the psychiatric symptoms were not prominent and there was no evidence of shunt malfunction, we suspended the use of antipsychotics and prescribed Madopar alone. The dose of Madopar was increased to 1250 mg per day, and over the next five days, the patient became flexible and able to speak. Upon discharge, he was still suffering from parkinsonism and presented with Parinaud syndrome, a supranuclear paralysis of vertical gaze resulting from damage to the mesencephalon [3], but he could eat and walk with help. After being treated with Madopar (1250 mg/d) for one month, the patient was able to care for himself and was fully mobile. As a result, he stopped Madopar, and his parkinsonism did not relapse within one month after discontinuation of therapy.

**Fig. 1:**
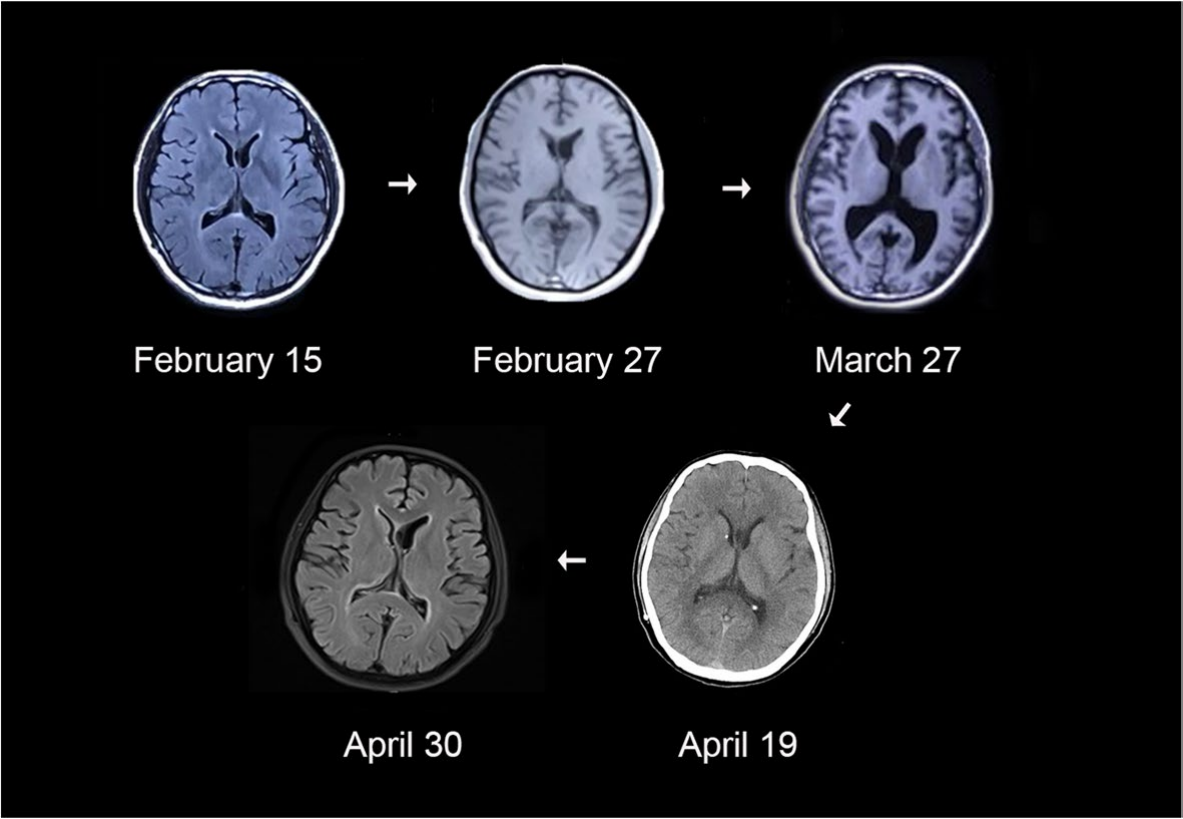
Serial axial MRI and CT scans showing that the ventricles changed significantly from normal to enlarged and then back to normal, showing the fluctuation of the lateral ventricle

The results of CSF examination were normal (Table 1). Brain magnetic resonance imaging (MRI) and computed tomography (CT) scans displayed fluctuations in the lateral ventricle (Fig. 1).

**Table 1:** Summary of the cerebrospinal fluid tests

Test items	Results		Reference range	
	25 August 2016	26 April 2021		
Pressure	140 mmH_2_O	150 mmH_2_O	80 mmH_2_O-180 mmH_2_O	
Total cell count		2005 × 10^6^/L	(0–8)×10^6^/L	
Leukocyte count	0	5 × 10^6^/L	0 × 10^6^/L	
Protein	22.1 mg/dL	48.60 mg/dL	15–45 mg/dL	
Glucose	61.79 mg/dL	54.18 mg/dL	45–80 mg/dL	
Immunoglobulin G		9.56 mg/dl	7.51–15.6 g/L	
Immunoglobulin A		0.59 mg/dl	0.82–4.53 g/L	
Immunoglobulin M		0.34 mg/dl	0.46–3.04 g/L	
Antibodies against NMDARs, LGI1	negative	negative	negative	
Antibodies against Hu (e1), Yo (PCA1), and Ri (ANNA2)		negative	negative	
Metagenomic next-generation sequencing		negative	negative	
Xpert	negative	negative	negative	
TB-spot	negative	negative	negative	
Cysticercosis	negative	negative	negative	

*NMDARs* N-methyl-D-aspartate receptors, *LGI1* Leucine-rich glioma-inactivated 1

The patient had no related previous physical or mental illness, no toxic exposure and no medical history. Furthermore, no other family members had a similar history.

## Discussion and conclusions

In our case, parkinsonism developed prior to the use of antipsychotics and gradually progressed until April 19, 2021, when the patient was admitted to our hospital. At that time, his symptoms peaked. However, there was no artificial adjustment of the shunt device, and CT showed that the lateral ventricle and the third ventricle had returned to normal levels. In summary, the head imaging results did not match the clinical symptoms, and the patient had a good response to high-dose Madopar.

We faced a huge challenge in the diagnosis and treatment of this patient, and the differential diagnosis may include several considerations. Parkinson’s disease is characteristic by bradykinesia in combination, with either rest tremor and rigidity. However, our patient presented as downward vertical supranuclear gaze palsy, which is the absolute exclusion criteria for Parkinson’s disease [4], so this diagnosis was ruled out. Progressive supranuclear palsy (PSP) should also be differentiated. PSP is a neurological syndrome characterized by movement disorders, speech disorders and behavioral abnormalities. The disease progresses slowly and has a limited response to Madopar [5], which is not consistent with the actual course of our patient. Normlpressurehydrocephlus is another disease that needs to be considered. These patients present parkinsonism and cognitive impairment. Head MRI and CT showed the ventricles were enlarged. The pressure of CSF was normal. However, our patient didn’t have urination disorders, and ventricular automatic back to normal size without shunt malfunction and any interventions [6], so we didn’t consider the diagnosis. In addition, viral encephalitis presented with disturbance of consciousness and cognitive impairment, so we should consider it. However, no abnormality was found in the metagenomics next generation sequencing, and antiviral treatment in other hospitals was ineffective. Therefore, we excluded this diagnosis. Young patients with consciousness impairment and cognitive impairment need to be considered of autoimmune encephalitis, high-dose hormone is invalid, and the result of autoimmune encephalitis antibody was negative, so the disease was not considered. Therefore, we may consider parkinsonism after VP shunt for hydrocephalus.

**Fig. 2:**
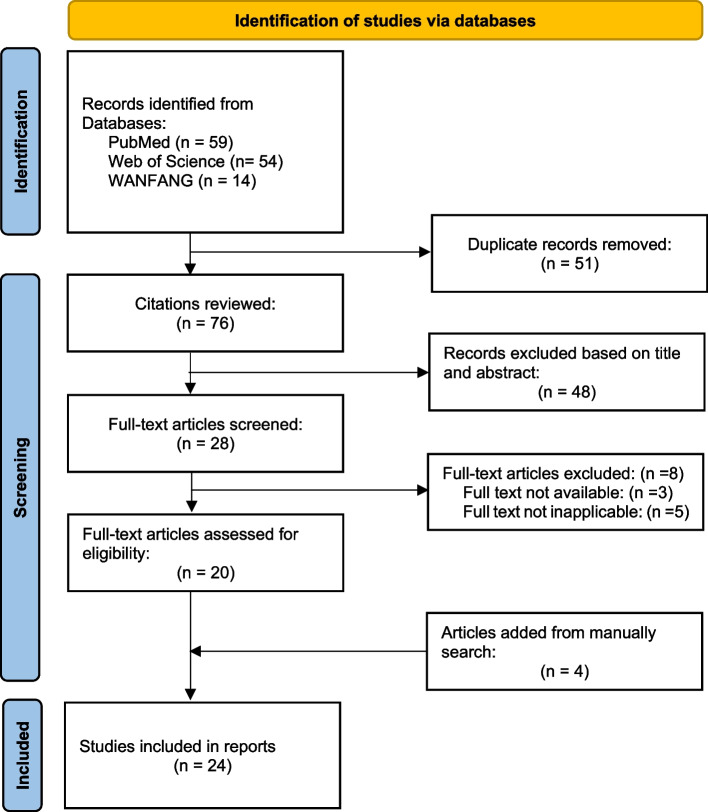
Flow chart of the systematic search and study selection process

We searched the PubMed, Web of Science, and WANFANG databases with Medical Subject Headings (MeSH) index terms. Search results were merged using reference management software, and any duplicate records of the same report were removed. Among the identified records, this review included only those whose participants (1) underwent single or multiple VP shunt due to hydrocephalus and (2) had parkinsonian symptoms. Studies that clearly did not meet the initial criteria were rejected on initial review. Reviews, conference papers, abstracts without available full text and studies written in languages other than English or Chinese were also excluded. Ultimately, this literature review included 24 articles that reported parkinsonism after VP shunt for hydrocephalus in 38 patients (Fig. 2; Table 2) [7–30]. These patients comprised 26 males and 12 females whose age at the onset of parkinsonism ranged from 7 to 72 years. The reported preoperative initial diagnosis was hydrocephalus in all cases; the aetiological type was mostly obstructive hydrocephalus caused by midbrain aqueduct stenosis (22/38), tumours (4/38), or other causes (12/38). After single (19/38) or multiple (19/38) VP shunt placement, these patients’ symptoms of hydrocephalus were improved significantly. The time between the completion of surgery and the onset of parkinsonism ranged from less than 1 day to 16 years. Patients presented with stiff facial expressions, salivation, dysphagia, low voice, limb tremor, stiffness, and bradykinesia. Neurological examination revealed Parinaud syndrome, cognitive impairment, tendon hyperreflexia, and positive bilateral pyramidal tract signs. Imaging showed fluctuating changes in the lateral ventricle and the third ventricle without any obstruction of or other interference with the shunt device. In addition, reduced uptake of radionuclides could be seen in the striatum or the frontal cortex.

**Table 2:** Summary of clinical features in patients with Parkinsonism following VP shunt due to hydrocephalus

Case n°	Author(s)/year	Age (yrs)/sex	Hydrocephalus (HD)	Operation	Time of onset	Treatment	Prognosis/Time
1	Berger et al., 1985 [8]	21/F	obstructive HD due to AS	multiple shunt revisions	2 weeks after the first revision	another shunt revision, Cogentin 2 mg TID, Prolopa 100 mg BID	improved following this revision; motor examination was normal on medications/6 months
2	Curran et al., 1994 [9]	16/B	obstructive HD due to AS	multiple shunt revisions	after the last revision	levodopa/carbidopa	parkinsonism improved
3		21/M	obstructive hydrocephalus secondary to a pineal mass	VP shunt	1 year after VP shunt	levodopa	parkinsonism improved/1 year
4		7/B	obstructive HD due to AS	multiple shunt revisions	after the last revision	levodopa	parkinsonism improved
5		72/M	normal pressure hydrocephalus	VP shunt	4 years after VP shunt	levodopa	parkinsonism improved
6	Keane et al., 1995 [11]	32/F	obstructive hydrocephalus caused by cysticercosis	multiple shunt revisions	unknown	placement of a shunt with a low-pressure valve	extrapyramidal signs became less prominent
7	Shahar et al., 1998 [16]	17/B	obstructive HD due to AS	multiple shunt revisions	2 weeks after the last revision	levodopa/carbidopa 100/25 mg TID	remarkable improvement/1 week
8	Asamoto et al., 1998 [7]	18/F	obstructive HD due to AS	VP shunt	developed acute parkinsonism after the shunt revision	levodopa	parkinsonism improved
9	Zeidler et al., 1998 [24]	57/M	obstructive HD due to AS	VP shunt, subsequent shunt replacement failure, Torkildsen operation	16 months after the last operation	bromocriptine 3 mg BID, Sinemet Plus 2 tablets QID	extrapyramidal signs becoming less prominent
10		21/F	obstructive HD due to AS	multiple shunt revisions	3 months after the last revision	Madopar 125 mg to 250 mg TID	improvement in rigidity/2 days; spontaneous speech and increased mobility/2 weeks
11	Ochiai et al., 2000 [14]	59/M	obstructive HD	multiple shunt revisions	3 months after the last revision	bromocriptine/amantadine, L-dopa	bromocriptine/amantadine had no effect; the parkinsonism dramatically improved with administration of L-dopa
12		32/M	obstructive HD	multiple shunt revisions	3 months after the last revision	bromocriptine/amantadine, L-dopa	bromocriptine/amantadine had no effect; the parkinsonism dramatically improved with administration of L-dopa
13	Sun et al., 2001 [28]	19/M	obstructive HD	VP shunt	4 months after VP shunt	amantadine/Artane	treatment was ineffective; patient died/1 year
14		14/B	obstructive HD	VP shunt	2 months after VP shunt	amantadine/Artane	parkinsonian features improved/unknown
15		21/M	communicating hydrocephalus	VP shunt	3 months after VP shunt	pressure shunt pump	parkinsonian features improved, but patient died of leukaemia/unknown
16		18/M	obstructive HD due to AS	VP shunt	4 months after VP shunt	niacin, amantadine/Artane	parkinsonian features improved/3 days
17		27/F	obstructive HD	VP shunt	2 years after VP shunt	amantadine/Artane, shunt revisions	parkinsonism presented upon use of medication/unknown
18	Tokunaga et al., 2003 [22]	26/M	obstructive hydrocephalus after possible encephalitis	VP shunt	8 months after VP shunt	endoscopic third ventriculostomy	patient became capable of walking/2 months
19	Racette et al., 2004 [17]	44/M	obstructive HD due to AS	VP shunt and shunt revisions	10 days after revision	levodopa/carbidopa 1500/375 mg QD	improved verbal response time, bradykinesia, and rigidity/unknown
20	Yomo et al., 2006 [23]	64/M	obstructive HD due to AS	VP shunt multiple shunt revisions	4 months after the VP shunt	levodopa/carbidopa 300/30 mg QD to 600/60 mg QD	parkinsonism presented after several months because of slit ventricle syndrome
21	Kim et al., 2006 [12]	46/M	obstructive HD due to AS	multiple shunt revisions	1 week after the second revision	levodopa/carbidopa 100/25 mg TID	parkinsonian features markedly improved/3 days
22	Prashantha et al., 2008 [16]	38/M	obstructive HD due to AS	multiple shunt revisions	3 days after the last revision	levodopa/carbidopa, total dose 275 mg QID	good response to levodopa/3 weeks; almost asymptomatic/3 months
23	Kinugawa et al., 2009 [13]	49/M	obstructive HD due to AS	VP shunt	3 months after VP shunt	levodopa 300 mg QD trihexyphenidyl 6 mg QD	parkinsonism improved but fully recurred/1 year
24	Sakurai et al., 2010 [19]	46/F	obstructive HD due to AS	VP shunt	unknown	levodopa 600 mg QD	parkinsonian features markedly improved/unknown
25	Hashizume et al., 2011 [10]	47/F	obstructive HD due to AS	VP shunt	1 year after VP shunt	levodopa/carbidopa 1000/100 mg QD endoscopic third ventriculostomy	no improvement with medication; symptoms improved 2 months after surgery
26	Lau et al., 2011 [30]	17/B	obstructive HD due to pineal tumour	VP shunt	3 years after VP shunt	acute shunt malfunction	total recovery after a month
27	Rebai et al., 2012 [18]	11/F	AS due to tectal tumour	ETV failure, multiple shunt revisions	1 day after the last revision	levodopa 125 mg TID bromocriptine 30 mg QD	regained the ability to stand and walk with assistance/2 weeks
28	Okawa et al., 2015 [15]	51/M	obstructive HD due to AS after the bleeding in the fourth ventricle surgery	VP shunt	2 months after VP shunt	levodopa/benserazide 1200/300 mg QD endoscopic third ventriculostomy	preoperative medication for ETV was ineffective; patient was able to walk with a walker after ETV combined with 5 months of medication use
29	Li et al., 2017 [27]	41/M	obstructive HD due to AS	VP shunt	1 month after VP shunt	levodopa/benserazide 200/50 mg TID amantadine 100 mg TID	parkinsonian features markedly improved/10 days
30		35/F	hydrocephalus caused by traumatic brain injury	VP shunt	16 years after VP shunt	levodopa/benserazide 400/100 mg TID	parkinsonian features markedly improved/25 days
31		18/F	hydrocephalus caused by traumatic brain injury	VP shunt	5 months after VP shunt	levodopa/benserazide 100/25 mg TID	could eat independently, increased physical activity/1 month
32	Zhou et al., 2019 [25]	45/M	obstructive HD due to AS	VP shunt and an increase in pressure	6 months after the last revision	Madopar/pramipexole 125/0.125 mg TID for 10 days, Madopar 250 mg TID for two years	parkinsonian features started to improve/10 days; parkinsonian features did not recur/2-year follow-up period
33	Shpiner et al., 2021 [21]	35/M	obstructive HD due to AS	multiple recalibrations	3 weeks after the last recalibration	carbidopa/levodopa 187.5/750 mg QD, endoscopic third ventriculostomy	presented with parkinsonism/unknown
34		26/M	obstructive HD due to pineal tumour	multiple shunt revisions	6 months after the last revision	carbidopa/levodopa 75/300 mg QD	presented with parkinsonism /10 days after medication use
35	Costa et al., 2021 [26]	38/M	obstructive HD due to AS	VP shunt	2 weeks after VP shunt	levodopa/benserazide 250 mg QID, bromocriptine 5 mg TID	good control established in 6 months
36	Villamil et al.,2022 [29]	42/F	obstructive HD due to AS	endoscopic third ventriculostomy and VP shunt	2 months after VP shunt	levodopa-carbidopa 250/25 mg QID	after 2 years of follow-up, patient remained on chronic treatment with L-dopa and was responding well
37		32/F	obstructive HD due to rosette-forming glioneuronal tumour	endoscopic third ventriculostomy, VP shunt and shunt revision	4 months after the last revision	levodopa-carbidopa 250/25 mg QID	good control established in 6 months
38		20/M	obstructive HD due to AS	multiple shunt revisions	3 months after the last revision	cabergoline 10 mg QD, amantadine 100 mg TID	completely resolved in 2 months

*HD* Hydrocephalus, *AS* Aqueductal stenosis, *VP* Ventriculoperitoneal, *QD* Quaque die, *BID* Bis in die, *TID* Ter in die; mg = milligrams

The pathogenesis of parkinsonism secondary to VP shunt has not been clarified. In current thinking, the widely accepted mechanisms involve two main pathways. One is reversible dysfunction of the presynaptic nigrostriatal dopaminergic pathway. 6-[18 F] Fluorodopa positron emission tomography (PET) imaging has shown reduced uptake in the striatum [31], indicating that the striatum is directly damaged, the substantia nigra striatum pathway is interrupted, or the substantia nigra pars compacta is dysfunctional [13]. 6-[18 F] Fluorodopa PET imaging has thus provided important evidence of the pathophysiology of parkinsonism secondary to VP shunt. First, the enlarged lateral and third ventricles directly constrict the caudate, leading to the impairment of blood flow or neuronal transport in the striatum, which results in dopamine deficiency [24]. A single-photon emission computed tomography (SPECT) study showed reduced cerebral blood flow in the bilateral caudate, further supporting this hypothesis [32]. Second, some authors have suggested that the brain undergoes a series of expansion and contraction cycles; therefore, repeated cycles of ventricular dilatation and relaxation may somehow alter their physical characteristics and increase the speed of dilatation in response to increased intracranial pressure, leading to damage [24]. The medial aspects of the substantia nigra pars compacta are more easily affected by increased intracranial pressure than other parts, resulting in a significant loss of dopamine neurons and a reduction in dopamine release [33]. Furthermore, some authors have proposed that the effect of shear forces from fluctuations in the lateral ventricle causes mechanical disruption of nigrostriatal projection fibres [16].

The other mechanism is cortico-basal ganglia loop dysfunction. Interestingly, in a few cases, 99mTc-ECD SPECT and [18 F] DOPA PET revealed severely low uptake in the frontal lobe cortex but not in the striatum [34], implying the impairment of more distal basal ganglia connections [9]. Researchers have found that some cases of parkinsonism are related to cortico-basal ganglia loop dysfunction resulting from reduced cerebral cortical blood flow without a loss of nigrostriatal dopaminergic neurons [10, 35]. Additionally, scholars propose that artificial alteration of the transtentorial pressure balance results in hydrocephalus and excessive drainage, which directly damage the periaqueductal grey, or that anatomical midbrain deformities can be associated with chronic interruption of mesencephalic blood flow [24]. While, this type of parkinsonism is caused by frontal lobe dysfunction resulting from midbrain dysfunction. Furthermore, previous studies showed corpus callosum hyperintensity in patients with hydrocephalus after VP shunt [36], and based on this finding, some authors believe that impingement on the corpus callosum by the rigid falx or stretching of the corpus callosum and pericallosal artery by the dilatation of the lateral ventricles may cause axonal degeneration or vascular insufficiency of the corpus callosum and result in atrophy of the frontal cortical regions [23].

Two treatments for parkinsonism after VP shunt have been used in the literature: levodopa and ETV. According to the physiopathology mentioned above, if the presynaptic nigrostriatal dopaminergic pathway is involved, the patient should have a positive response to levodopa. In previous case reports, 28 patients (28/38) were treated with high-dose levodopa with a maximum dose of 1500 mg/d [7, 9, 10, 12–21, 23–26, 29], and combination treatments such as amantadine and bromocriptine were used in 17 patients [8, 13–15, 18, 24–26, 29]. Twenty-three patients (23/28) who were on levodopa had significant positive effects after 3 days to 6 months of treatment; their clinical symptoms were obviously improved, the dose of levodopa could be reduced slowly after 1 year, and no recurrence was observed after the drug therapy was stopped [7, 9, 12–14, 16–20, 23–27, 29].

Conversely, when levodopa has a poor effect or the cortico-basal ganglia loop is involved, ETV has been shown to significantly improve parkinsonism by restoring blood supply to allow adequate dopamine uptake in the brain’s frontal cortex [10]. Five cases were reported to have poor responses to high-dose dopaminergic drugs and no improvement after longer-term treatment with larger doses; this resistance suggests that parkinsonism could also arise from the disturbance of more distal connections than the presynaptic nigrostriatal dopaminergic pathway in the cortico-basal ganglia loop. PET results support this hypothesis, and ETV treatment has resulted in improved prognoses [10, 15, 21, 22]. In some patients, ETV to improve symptoms should be selected as soon as possible after the occurrence of slit ventricle syndrome following VP shunt, and shunt revision and levodopa therapy are usually ineffective [23]. Some authors have proposed that ETV alone can lead to complete remission of global rostral midbrain dysfunction. Another option for treatment is levodopa administration after ETV [10, 15]. However, there is no clear consensus on this matter, and further study is warranted.

Our patient was diagnosed with parkinsonism after VP shunt due to presynaptic nigrostriatal dopaminergic pathway impairment. Interestingly, his symptoms were significantly aggravated by antipsychotic medications, which could be explained by a “two-hit” mechanism. Fluctuations in the lateral ventricle represented the first hit to the presynaptic nigrostriatal dopaminergic pathway, and antipsychotic drugs had an antagonistic effect on dopamine D2 receptors, providing the second hit; thus, the patient’s clinical symptoms were rapidly aggravated. This situation has rarely been seen in cases of patients with parkinsonism after VP shunt reported previously. Therefore, our report provides clinicians with an important reminder: patients with parkinsonism after VP shunt are not suitable candidates to receive dopamine D2 receptor blockers.

Unfortunately, our patient was not evaluated for DAT/VMAT2 PET (at present, DAT/VMAT2 PET replaces 18 F-DOPA PET) because of financial constraints. However, the good efficacy of high-dose Madopar supported the mechanistic hypothesis described above.

## Data Availability

Data sharing is not applicable to this article as no datasets were generated or analyzed.

## Abbreviations

VP: ventriculoperitoneal
ETV: endoscopic third ventriculostomy
mg: milligrams
CSF: cerebrospinal fluid
MRI: magnetic resonance imaging
CT: computed tomography
PET: positron emission tomography
SPECT: single-photon emission computed tomography.
